# Early Cognitive/Social Deficits and Late Motor Phenotype in Conditional Wild-Type TDP-43 Transgenic Mice

**DOI:** 10.3389/fnagi.2016.00310

**Published:** 2016-12-20

**Authors:** Julio A. Alfieri, Pablo R. Silva, Lionel M. Igaz

**Affiliations:** IFIBIO Houssay, Grupo de Neurociencia de Sistemas, Facultad de Medicina, Universidad de Buenos Aires - CONICETBuenos Aires, Argentina

**Keywords:** TDP-43, frontotemporal dementia, amyotrophic lateral sclerosis, transgenic mice, behavior, animal model, proteinopathy

## Abstract

Frontotemporal Dementia (FTD) and amyotrophic lateral sclerosis (ALS) are two neurodegenerative diseases associated to mislocalization and aggregation of TAR DNA-binding protein 43 (TDP-43). To investigate in depth the behavioral phenotype associated with this proteinopathy, we used as a model transgenic (Tg) mice conditionally overexpressing human wild-type TDP 43 protein (hTDP-43-WT) in forebrain neurons. We previously characterized these mice at the neuropathological level and found progressive neurodegeneration and other features that evoke human TDP-43 proteinopathies of the FTD/ALS spectrum. In the present study we analyzed the behavior of mice at multiple domains, including motor, social and cognitive performance. Our results indicate that young hTDP-43-WT Tg mice (1 month after post-weaning transgene induction) present a normal motor phenotype compared to control littermates, as assessed by accelerated rotarod performance, spontaneous locomotor activity in the open field test and a mild degree of spasticity shown by a clasping phenotype. Analysis of social and cognitive behavior showed a rapid installment of deficits in social interaction, working memory (Y-maze test) and recognition memory (novel object recognition test) in the absence of overt motor abnormalities. To investigate if the motor phenotype worsen with age, we analyzed the behavior of mice after long-term (up to 12 months) transgene induction. Our results reveal a decreased performance on the rotarod test and in the hanging wire test, indicating a motor phenotype that was absent in younger mice. In addition, long-term hTDP-43-WT expression led to hyperlocomotion in the open field test. In sum, these results demonstrate a time-dependent emergence of a motor phenotype in older hTDP-43-WT Tg mice, recapitulating aspects of clinical FTD presentations with motor involvement in human patients, and providing a complementary animal model for studying TDP-43 proteinopathies.

## Introduction

Many neurodegenerative diseases are associated with characteristic changes in the behavioral profile of affected individuals. For example, frontotemporal dementia (FTD) comprises a group of clinical syndromes unified by underlying frontotemporal lobar degeneration (FTLD) pathology, which leads to disorders of behavior, language and executive function (Woollacott and Rohrer, 2016). FTD is the second most common form of dementia after Alzheimer’s disease in those under 65 years of age, and is characterized by progressive degeneration of frontal and anterior temporal lobes. In amyotrophic lateral sclerosis (ALS), a fatal neurodegenerative disorder that progressively affects upper and lower motor neurons, the primary symptoms are associated with motor function deficits (Morris, 2015). Recently, there has been a growing body of literature demonstrating a clinical and neuropathological overlap between FTD and ALS, disorders which can be viewed as representations of the extremes of a disease spectrum (Ferrari et al., 2011).

Several neurodegenerative diseases display TAR DNA-binding protein 43 (TDP-43) pathology and this protein was identified as the main component of the distinctive cytoplasmic aggregates seen in the vast majority of ALS cases and about half of the cases of FTD (FTLD-TDP; Neumann et al., 2006; Baralle et al., 2013). These and other neurodegenerative disorders with the presence of aggregated TDP-43 are now collectively referred to as “TDP-43 proteinopathies” (Kwong et al., 2008). Although it is clearly not possible to faithfully model every clinicopathological feature of the FTD/ALS spectrum in rodents, transgenic (Tg) mice have been shown to recapitulate major aspects of human FTD and ALS. These include animal models based on the genetic manipulation of Tau, TDP-43, SOD1 and C9ORF72, among others, each one displaying specific but partial features of these diseases (Roberson, 2012; Philips and Rothstein, 2015).

Behavioral phenotyping has been particularly challenging since these models often display multiple abnormalities, which can be heavily influenced by the gene, mutation and/or promoter used for the genetic manipulation (Vernay et al., 2016a). In particular, TDP-43 mouse models have been rapidly developed over the last few years, usually showing clear signs of neurodegeneration and other histopathological changes typical of the human disease (Liu et al., 2013; Picher-Martel et al., 2016). In terms of behavioral abnormalities, most TDP-43 rodent models display early motor deficits, frequently associated with the use of pan-neuronal, constitutive promoters affecting developmental milestones or proper motor function, conceivably masking other phenotypic domains. A subset of models have used promoters that allow restricted expression in terms of timing and/or cellular subgrouping. Among these, we have previously developed and characterized TDP-43 Tg mice with inducible, forebrain enriched neuronal expression using a CamKIIα promoter coupled to a tTA system (Igaz et al., 2011). These mice express either nuclear (TDP-43-WT) or cytoplasmic (TDP-43-ΔNLS) forms of the protein, and they recapitulated several aspects of TDP-43 proteinopathies, including time-dependent neuronal loss, gliosis, corticospinal tract degeneration and global changes in gene expression (Igaz et al., 2011). Subsequent in-depth behavioral analysis of inducible TDP-43-ΔNLS mice demonstrated an early establishment of motor, cognitive and social abnormalities (Alfieri et al., 2014), of relevance since these three behavioral domains are affected in patients with different presentations within the clinicopathological spectrum of FTD/ALS (Giordana et al., 2011; Seltman and Matthews, 2012; Gordon, 2013).

In this work, we sought to thoroughly study the behavioral changes in our inducible TDP-43-WT mouse model. Post-weaning 1 month induction of the Tg caused early deficits in social behavior, a characteristic early symptom of FTD patients (Shinagawa et al., 2006; Harciarek and Cosentino, 2013). A battery of cognitive tasks revealed early alterations in object recognition and working memory tests, while aversive memory was spared. Remarkably, motor tests demonstrated preserved function in the open field, rotarod and hanging wire tests at 1 month of induction, with only a mild clasping phenotype. Finally, we studied the time course of motor behavior up to 12 months of Tg induction and showed a gradual, time-dependent installment of motor phenotypes as evidenced by impaired performance in the rotarod and hanging wire test, and emergence of hyperlocomotion in the open field test. These results reveal different sensitivities to TDP-43-WT expression in the brain circuits underlying social/cognitive and motor behavior.

## Materials and Methods

### Animals

The experimental protocol for this study was approved by the National Animal Care and Use Committee of the University of Buenos Aires (CICUAL). hTDP-43 Tg lines were generated by injection of linearized moPrP-tetP vector containing human wild-type TDP 43 protein (hTDP-43-WT) cDNA into pronucleus of fertilized eggs from C57BL/6J × C3HeJ F1 matings. Monogenic tetO-TDP-WT12 mice were bred to Camk2a-tTA mice (Mayford et al., 1996; Jackson Laboratory) generating non-Tg (nTg), tTA monogenic, single tetO-TDP-43 Tg mice (non-TDP-43 expressing control mice) and bigenic mice expressing hTDP-43-WT12 (hereinafter referred to as tTA/WT12).

**Figure 1:**
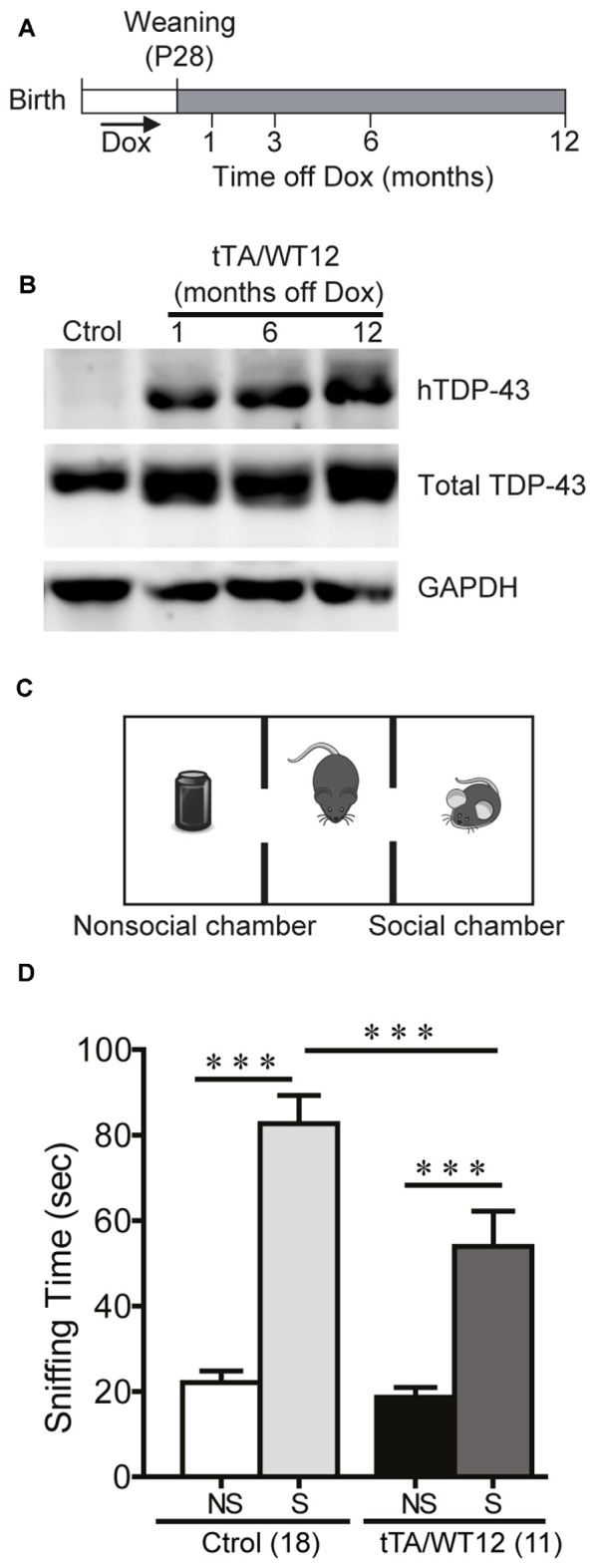
**Altered social behavior in TDP-43-WT transgenic (Tg) mice. (A)** Experimental design: transgene expression was activated at weaning (postnatal day 28) by removing Dox from water. The behavioral responses of these Tg mice were analyzed at the indicated time points after weaning. **(B)** Expression of human TAR DNA-binding protein 43 (TDP-43) in Tg mice. Immunoblot of hTDP-43 or total TDP-43 (h+mTDP-43) in cortical RIPA extracts of control (non-Tg) and tTA/WT12 (1, 6 or 12 months off Dox) mice. GAPDH is a loading control. **(C)** Schematic view of the three-chamber social interaction apparatus, consisting of a black Plexiglas rectangular box with three interconnected chambers. **(D)** Time spent sniffing the social (S; P21-P28 mouse) or the non–social (NS; black plastic object) stimulus during a 10 min session (test phase) was recorded. 1 month off Dox bigenic mice (tTA/WT12) presented a reduced social interaction time during the session (***p < 0.001, one-way ANOVA/Newman-Keuls *post hoc* test). Number of animals is indicated in parentheses. The data represent mean ± SEM.

Breeding mice and pups were treated with 0.2 mg/ml Dox (Doxycycline Hyclate, sc-204734A, Santa Cruz Biotechnology) in drinking water, in order to circumvent potential prenatal and postnatal developmental effects of Tg expression. Induction of hTDP-43 was achieved by switching mice to regular drinking water (without Dox) at weaning (postnatal day 28) and mice were analyzed at different time points (Figure 1A).

Genomic DNA isolated from ear biopsies were screened for the presence of the Tg by means of PCR amplification with the following primers: TDP-forward (TTGGTAATAGCAGAGGGGGTGGAG), MoPrP-reverse (TCCCCCAGCCTAGACCACGAGAAT), Camk2a-tTA-forward (CGCTGTGGGGCATTTTACTTTAG) and Camk2a-tTA-reverse (CATGTCCAGATCGAAATCGTC) as previously described (Igaz et al., 2011; Alfieri et al., 2014). The TDP-43-WT12 Tg line used in these experiments was established by crossbreeding with C57BL/6J mice for 7–8 generations to homogenize genetic background and minimize variability. Both Tg and control animals of either sex were included in the experiments performed in the different age groups studied.

### Behavioral Studies

Mice were kept on a 12 h light/dark cycle under controlled conditions of temperature (23 ± 2°C) and humidity (40%–60%), with *ad libitum* access to food and water. All behavioral tasks were performed during the light phase (lights on 7 am; lights off 7 pm) with the exception of the Y-maze spontaneous alternation, which was conducted during the initial dark phase (7:00 p.m. to 9:00 p.m.) to maximize exploratory behavior and consistently obtain a high number of entries. All sessions were video recorded through a camera mounted above the arena (unless noticed) and mouse position was determined by automatic video tracking (ANY-maze, Stoelting Co). The animals were allowed to habituate in the experimental room (with attenuated light and sound) for at least 1 h prior to the tests. The objects, floor and walls of the mazes used in behavioral analysis were cleaned with ethanol 10% between sessions.

Similarly to what we previously demonstrated in experiments with TDP-43-NLS Tg mice (Alfieri et al., 2014), all non-bigenic offspring (nTg and both single Tg mice) exhibited similar behavioral responses. Thus, for all subsequent behavioral tests and other experimental analysis we grouped these genotypes under the Control group to compare against the Bigenic mice.

#### Social Interaction Test

This task was performed on a three-chamber box as previously described (Brodkin et al., 2004; Alfieri et al., 2014). Briefly, the test apparatus consisted of a black Plexiglas rectangular box (40.6 cm × 15 cm × 23 cm) with three interconnected chambers, placed under dim light (25 lux). Prior to the start of each test, one of the end chambers was randomly designated the “nonsocial side” and the other the “social side”. During the habituation phase, two clear Plexiglas cylinders with multiple holes were placed in the apparatus, one in each end chamber. Animals (test mice) were placed in the central chamber and allowed to explore the whole apparatus during 5 min. After habituation a stimulus mouse (21–28 days old C57BL/6J male mouse) was placed into the cylinder on the side that had been designated the “social side” and a black plastic object was placed into the cylinder on the “nonsocial side” (nonsocial stimulus). Mice have a natural tendency to explore a novel conspecific over a novel object. The test mouse was able to freely explore the apparatus for 10 min (test phase). Time spent sniffing the social and nonsocial stimuli and time spent in each chamber was measured. Clean bedding was placed on the apparatus floor prior to the next test.

#### Novel Object Recognition

To analyze the impact of TDP-43-WT12 expression on recognition memory, we used the novel object recognition test. During habituation sessions, animals were introduced into an empty Plexiglas arena (40 cm × 23 cm × 15 cm) for 10 min during the first session (day 1) and two 5 min sessions during the second day. On the third day (training) the mice were exposed to two identical objects placed at opposite ends of the arena for 10 min. On test day (24 h later), the mice were allowed to explore one copy of the previously presented object (familiar) and a new object (novel) for 5 min. The time spent exploring the two objects was scored by an experimenter using the video recorded sessions. Exploration was defined as pointing the head toward an object at a distance of <2 cm from the object, with its neck extended and vibrissae moving. Turning around and sitting on the objects were not considered exploratory behaviors. The exploration time represents the percentage of time that mice spend exploring the object (familiar or novel) respect to the total exploration time (familiar + novel), as previously described (Alfieri et al., 2014).

#### Y-maze Spontaneous Alternation Test

A Y maze with three identical arms made of transparent Plexiglas (43 cm × 4 cm × 12.5 cm) placed at 120° angles to each other was used (Belforte et al., 2010; Alfieri et al., 2014) and placed in a room with clues to allow for visual orientation. Illumination was kept at 30 lux. Each mouse was placed at the end of one arm facing the center and allowed to explore the maze freely for 8 min without training, reward or punishment, while the experimenter remained out of sight. Entries into each arm were scored and alternation behavior was defined as a complete cycle of consecutive entrances into each of the three arms without repetition. The percentage of spontaneous alternation was defined as the number of actual alternations divided by the possible alternations [(# alternations)/(total arm entries − 2) × 100]. Total entries were scored as an index of ambulatory activity in the Y maze and mice with scores below 12 were excluded.

#### Step-Through Inhibitory (Passive) Avoidance

Inhibitory avoidance behavior was studied in a one-trial learning, step-through type situation which utilizes the natural preference of mice for a dark environment. The apparatus consists of two Plexiglas boxes (20 cm × 16 cm × 21 cm), one made of white Plexiglas and the other one of black Plexiglas, establishing two contiguous compartments connected through a sliding door. A stainless-steel grid floor spanned both compartments. The task was performed as previously described (Alfieri et al., 2014), with some modifications. Briefly, each mouse was placed during training in the light chamber for 60 s, then the door between the chambers was opened. The mouse received a footshock (0.2 mA, 50 Hz, 1 s) as it stepped into the dark compartment as described previously. Training latency was measured after door opening. Retention test was performed 24 h later. Each mouse was placed on the white compartment again (with the door open), and the step-through latency was recorded with a 300 s cutoff per session. In the retention test session the footshock was omitted.

#### Open Field

Assessment of general exploratory locomotion in a novel environment (open field test) was performed as previously described (Alfieri et al., 2014). Mice were placed in a clear Plexiglas (40 cm × 40 cm × 40 cm) arena with white floor divided into two zones: periphery and center (comprising 50% of the total area centered). Horizontal locomotor activity was assessed during 20 min. The open field arena was placed in the center of the room (1.8 m × 2 m) and illumination was kept at 50 lux. Total, peripheral and center distance traveled by the mice was quantified. Time bin analysis (every 5 min) was used.

#### Accelerated Rotarod

A rotarod apparatus (Ugo Basile, model 7600) was used to measure motor coordination and balance (Alfieri et al., 2014). For the accelerating rotarod test (4–40 rpm over 300 s) four trials per test were carried out during the test day, with a 2 min interval between trials. The latency to fall off the rotarod was recorded. Mice that rotated passively were scored as fallen.

#### Clasping Phenotype

hTDP-43-WT12 Tg mice as well as age-matched control mice were suspended by the tail 30 cm above an open cage for 30 s. Mice were slowly lowered toward the bottom of the cage, and a positive response was recorded for mice that clasped their limbs within 5 s of suspension while maintaining the clasping posture until lowered to the cage (Igaz et al., 2011; Alfieri et al., 2014).

#### Hanging Wire Grip Test

Grip strength was assessed using the hanging wire test, which was performed as described (Alfieri et al., 2014). Briefly, the mouse was placed on the top of a wire cage lid, then the lid was shaken lightly three times to cause the mouse to grip the wires and then the lid was turned upside down and held at a height approximately 20 cm above a cage containing fresh bedding. The latency to fall off the wire lid was quantified. A 60 s cutoff time was used.

#### Elevated Plus-Maze

Anxiety-like behavior was assessed as described (Braz et al., 2015) using an elevated plus maze consisting of two open arms (30 cm × 6 cm × 0.3 cm) and two closed arms (30 cm × 6 cm × 15 cm) with opaque walls. The apparatus was elevated 40 cm above the floor, and the duration of the test was 5 min. The maze was placed in the center of a homogenously illuminated room (2 m × 1.8 m; 100 lux across arms). At the beginning of the test, mice were placed in the central square facing the open arm opposite to the investigator. Number of open arm entries, percentage of time in open arms, total arm entries and total distance traveled was measured.

#### Visual Perception

Visual perception was evaluated in the visual cliff test as previously described (Alfieri et al., 2014). Briefly, a box with a simulated ledge was used and illumination was kept at 100 lux. The surface of the box (30 cm × 30 cm) and ledge (60 cm high) were covered with a black and white checkerboard pattern (2.5 cm × 2.5 cm) to emphasize the ledge dropoff. A piece of clear Plexiglas spans the ledge, resulting in the visual appearance of a cliff. The behavior was scored as “positive” when the mouse stopped at the virtual edge before attempting to cross. Visually impaired animals walk across the Plexiglas without stopping, and percentage of animals stopping at the edge was quantified. Vibrissae were removed to eliminate tactile placing responses.

### Brain Tissue Collection

After deep anesthesia with 5% chloral hydrate (1 ml/30 g), the mice were perfused transcardially with ice-cold PBS (0.1 M), pH 7.4 supplemented with 10 U/ml Heparin. The brains were immediately extracted and dissected into different areas including cerebral cortex, and then immediately frozen on dry ice and kept at −80°C for biochemical analysis.

### SDS-PAGE and Immunoblot Analysis

Tissues were extracted with 10 volumes (ml/g tissue) of RIPA buffer (0.1% SDS, 1% NP-40, 0.5% sodium deoxycholate, 5 mM EDTA, 150 mM NaCl, 50 mM Tris-HCl, pH 8.0) containing protease and phosphatase inhibitor cocktail (Roche), sonicated and centrifuged at 13,000 rpm for 20 min at 4°C. Protein concentrations were determined using the BCA assay kit (Pierce). Equal amounts (15 μg) of samples were subsequently resolved on 10% SDS-PAGE gels and transferred onto PVDF membranes (Immobilon-P, Millipore). Primary antibodies (described in Alfieri et al., 2014) were used as follows: rabbit anti-TDP-43 polyclonal antibody raised to amino acids 394–414 (1:20,000), human specific mouse anti-TDP-43 monoclonal antibody (60019-2, Proteintech, 1:2000) and anti-GAPDH mouse monoclonal antibody (6C5, Advanced ImmunoChemical Inc. 1:3000). Membranes were probed with corresponding secondary antibodies coupled to Alkaline Phosphatase and visualized with enhanced chemifluorescence (ECF, GE Healthcare Life Sciences). GAPDH was used as loading control. The blots were scanned in a Storm 845 PhosphorImager (GE Healthcare Life Sciences).

### Statistical Analysis

Data are expressed as mean values ± SEM and statistical analysis of behavioral tests was performed using PRISM 6 (Graph Pad software). Differences were considered to be significant when the probability value was <0.05. The following tests were used as required: Student’s *t* test (when comparing only two groups on one behavioral measure); one-way analysis of variance (ANOVA) followed by Newman-Keuls multiple comparison *post hoc* test (when comparing three or more groups); repeated measures ANOVA followed by Bonferroni’s multiple comparison *post hoc* test (for accelerated rotarod and open field time segment analysis); Fisher exact test (for clasping analysis). When nonparametric tests were required, Mann-Whitney test (for visual cliff test) and Kruskal-Wallis one-way ANOVA followed by individual Mann-Whitney *U* test (inhibitory avoidance task) were used.

## Results

In order to analyze the impact of forebrain specific, neuronal wild-type hTDP-43 expression in multiple behavioral domains, we used previously generated TDP-43 Tg mice with a tet-off system and the CaMKIIα promoter that model aspects of human TDP-43 proteinopathies (Igaz et al., 2011). Tg expression was induced at weaning and animals were analyzed at different times after Dox removal (Figure 1A). To assess proper induction of the Tg, we performed immunoblot analysis of human and total (human + mouse) TDP-43 in cortical RIPA fractions (Figure 1B), demonstrating robust and sustained expression of hTDP-43 as previously reported for the WT12 line (Igaz et al., 2011).

There were no signs of illness or alterations in the physical appearance of Tg mice (i.e., abnormal growth, posture and gait) that could potentially interfere with behavioral testing. Tg mice displayed normal righting reflex, and a previous analysis of forelimb and hindlimb striated muscles by H&E staining showed no evidence of muscle atrophy (Igaz et al., 2011). The body weight curve of bigenic mice up to 12 months off Dox showed no significant differences compared to control littermates (not shown). All behavioral tests were conducted around 1 month after induction (7–9 weeks old) unless otherwise stated.

### Conditional Overexpression of Human TDP-43 in Forebrain Neurons Leads to Impaired Social Behavior in Transgenic Mice

Social disinterest is observed as a recurrent feature of FTD patients (Shinagawa et al., 2006), and this behavior can be modeled in mice. We performed a variation of the three-chamber social interaction test (Figure 1C; Alfieri et al., 2014), which has been widely used in autism and FTD models (Yin et al., 2010; Gascon et al., 2014; Vernay et al., 2016a). This test measures the sociability of a mouse when given the choice between interacting with another mouse vs. an inanimate object. TDP-43-WT12 Tg mice showed a significant decrease in the exploration time of the social stimulus (another mouse) compared to control mice, while time exploring the nonsocial stimulus (object) was not different between groups (One-way ANOVA, *F*_(3,54)_ = 33.06, *p* < 0.0001; Figure 1D). Importantly, total exploration time in the social side showed non-significant differences, indicating that the decrease in social interaction time is not due to perception of the stimulus as aversive, increased anxiety-like behavior or decreased exploratory drive (254.4 ± 21.3 s vs. 201.3 ± 18.7 s for control and TDP-43-WT12 mice, respectively; *t*_(27)_ = 1.717, *p* = 0.0974). These results show that TDP-43-WT12 mice display sociability deficits.

### TDP-43-WT Mice Develop Cognitive Deficits

Although some information is available regarding cognitive performance in FTD/ALS animal models, including those based in TDP-43 manipulation, the information has been highly heterogeneous due to the use of different promoters and variants/mutations of the pathological protein (Philips and Rothstein, 2015; Vernay et al., 2016a). Since (1) Tg expression is enriched in forebrain neurons; and (2) high expression was observed in the hippocampus and cortex (Igaz et al., 2011), a battery of behavioral tests was used to determine whether the expression of human wild-type TDP-43 influences cognitive performance. Therefore, we set out to evaluate different aspects of cognitive function using novel object recognition, Y-maze and inhibitory avoidance tests.

**Figure 2:**
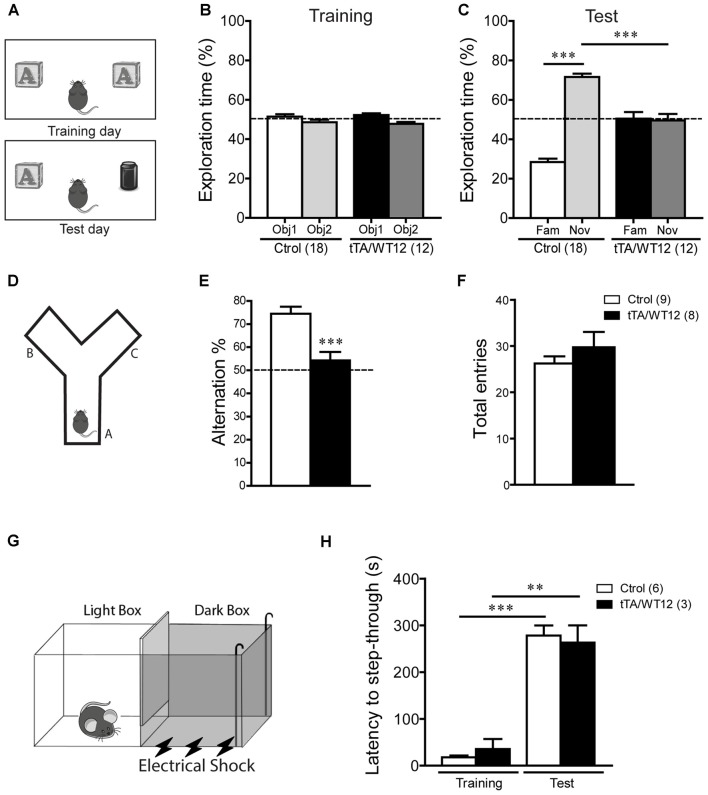
**Cognitive impairment in TDP-43-WT Tg mice. (A–C)** Novel object recognition test. **(A)** Scheme of training and test phases for the novel object recognition test. **(B)** Training day. Both control and TDP-43-WT Tg mice were exposed to two identical objects for 10 min and the time spent exploring each object was recorded. No significant differences in the exploration time (%) of the two objects were found in training phase. **(C)** Test day. 24 h after training, the recognition memory was measured while the animals were allowed to explore the familiar (Fam) and novel (Nov) objects for 5 min. The exploration time (%) represents the percentage of time that mice spend exploring the object (familiar or novel) respect to the total exploration time (familiar + novel). tTA/WT12 animals displayed a deficit in object recognition memory (***p < 0.001, one-way ANOVA/Newman-Keuls *post hoc* test). **(D–F)** Y-maze spontaneous alternation task. **(D)** Scheme of the Y-maze. **(E)** Mice were placed at the end of one arm facing the center and allowed to explore the maze freely for 8 min without training, reward or punishment. Entries into each arm were scored and alternation behavior was defined as a complete cycle of consecutive entrances into each of the three arms without repetition. Bigenic tTA/WT12 mice alternated between the arms at the chance (≈50%) level (***p < 0.001 significantly different from control group, Student’s *t* test; **F**) Total entries were scored as an index of locomotion activity in the Y maze. **(G,H)** Step-through inhibitory avoidance test. **(G)** Scheme of inhibitory avoidance apparatus. **(H)** During training, each mouse received a footshock (0.2 mA, 50 Hz, 1 s) as it stepped into the dark compartment. Retention test was performed 24 h later. The step-through latency was recorded; footshock was omitted during test session. No significant differences in latency values were found between controls and bigenic animals in either training or test phases, showing intact long term memory for this task (**p < 0.01, ***p < 0.001 Kruskal–Wallis one-way ANOVA followed by individual Mann-Whitney *U* test). Cognitive behavior was analyzed at 1 month off Dox. Number of animals is indicated in parentheses and data represent mean ± SEM.

We first used the novel object recognition test to assess recognition memory (Alfieri et al., 2014), a task that requires intact function of perirhinal and prefrontal cortices (Warburton and Brown, 2010; Figure 2A). In this test, where preference for a novel object is evaluated, both control and bigenic mice did not show a preference for any of two identical objects during the training day (Figure 2B). However, during test day (24 h later), novel object preference was clearly demonstrated in control mice but completely absent in TDP-43-WT Tg mice (One-way ANOVA, *F*_(3,56)_ = 66.0, *p* < 0.0001; Figure 2C). We next used the Y-maze spontaneous alternation test, a prefrontal cortex and hippocampal-dependent task (Lalonde, 2002), to assess spatial working memory (Figure 2D). While control animals display the expected alternation values avoiding the previously visited arms, bigenic mice alternated at chance (≈50%) level, indicating impaired working memory (*t*_(15)_ = 4.249 *p* = 0.0007; Figure 2E). Locomotion, estimated by the number of arm entries, was similar between groups (Figure 2F). Lastly, we performed the inhibitory avoidance task to study aversive memory (Figure 2G; Boccia et al., 2004; Alfieri et al., 2014). No difference in step-through latency was observed between groups during the acquisition (training) session, where mice were given a footshock upon entrance to the dark chamber. In a test session performed 24 h later, both bigenic and control animals displayed high latency values (close to the cut-off time), indicating preserved long-term memory for this task (Figure 2H). In summary, post-weaning neuronal TDP-43 overexpression impairs cognitive function in some but not all the paradigms tested.

### Preserved Motor Function in Young TDP-43-WT Mice

In the FTD/ALS spectrum of clinical presentations, motor deficits are a prominent feature of ALS but only a small fraction of FTD cases show motor abnormalities (Hodges et al., 2004; Seltman and Matthews, 2012). To evaluate this behavioral domain, we performed several tests, including open field, rotarod, hanging wire and clasping analysis.

**Figure 3:**
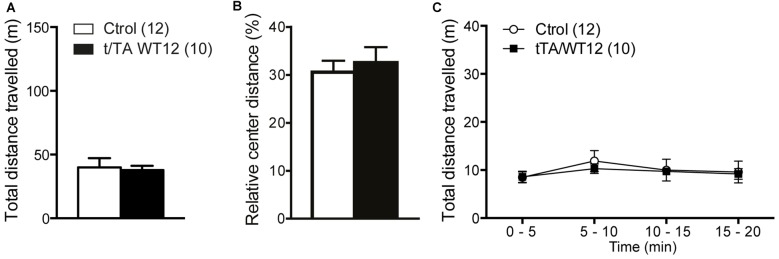
**Locomotor and exploratory behavior is preserved in young TDP-43-WT mice.** To measure general motor function and exploratory activity we used an open field test. Animals were placed in a novel environment during a 20 min session. **(A)** Total distance traveled, **(B)** relative center distance and **(C)** detailed measurement of total distance traveled in time segments of 5 min. No significant differences were found between controls and bigenic animals in locomotion or exploration (*p* > 0.05, Student’s *t* test for **A,B** or repeated-measures ANOVA in **C**). Number of animals is indicated in parentheses. Data represent mean ± SEM.

General motor function and exploratory activity can be evaluated using the open field test. Distance traveled during this task was similar between control and TDP-43-WT groups, suggesting no motor or exploratory abnormalities in our mouse model (Figure 3A). In addition, relative center distance was also unaffected (Figure 3B). To unmask any potential effect on locomotion not revealed by measuring total values, we divided the traveled distance in time bins and confirmed that this parameter was similar between genotypes across the whole duration of the test (Figure 3C).

**Figure 4:**
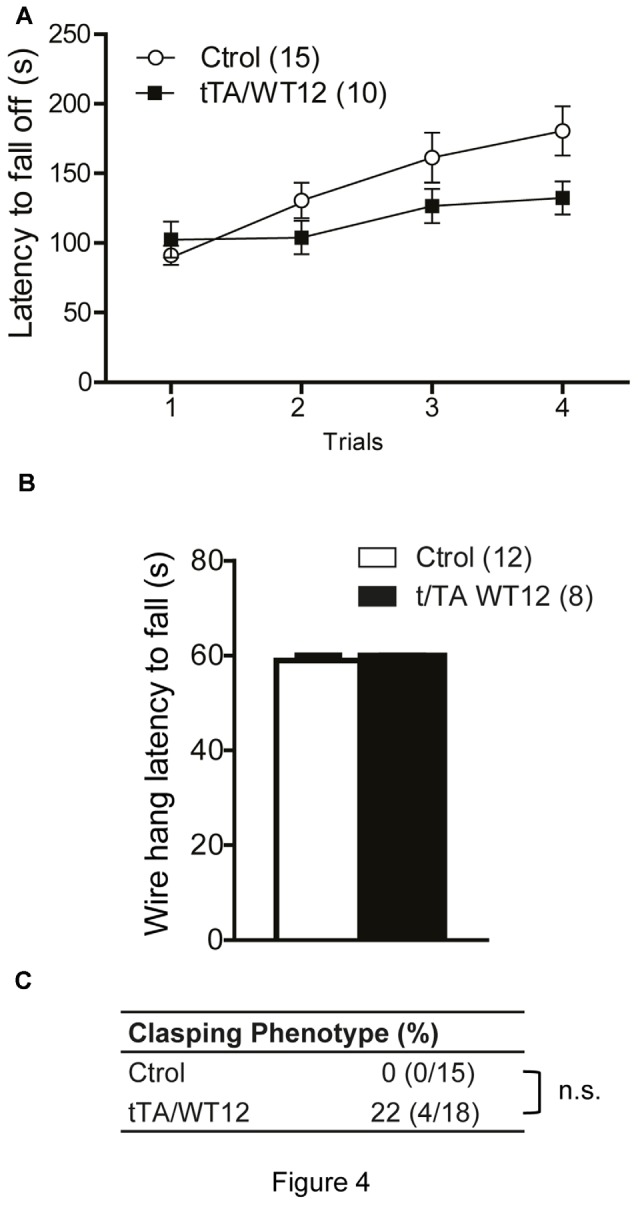
**Young TDP-43-WT Tg mice display normal motor coordination, balance and strength. (A)** Accelerated rotarod performance (4–40 rpm/5 min). Four trials per test were performed during the test day with a 2 min interval between trials. Latency to fall off the rotarod was recorded. **(B)** Hanging wire grip test. Grip strength was assessed using a standard wire cage turned upside down. The latency to fall off the wire lid was quantified. A 60 s cutoff time was used. No significant differences were found between control and bigenic animals (*p* > 0.05, repeated-measures ANOVA in **A**, Student’s *t* test in **B**). **(C)** Percentage of mice with clasping phenotype reveal mild incidence of spasticity, without reaching significance (Fischer Exact test, *p* > 0.05); n.s., non-significant differences respect to control group. Number of animals is indicated in parentheses. Data represent mean ± SEM.

The accelerated rotarod was used to assess motor coordination and balance. Both groups of mice behaved similarly, although there was a non-significant trend in bigenic mice towards impaired performance (repeated-measures ANOVA, *F*_(1,23)_ = 2.707, *p* = 0.1135 for group; *F*_(3,69)_ = 11.04, *p* < 0.0001 for trial; *F*_(3,69)_ = 2.498, *p* = 0.0668 for interaction; Figure 4A). TDP-43-WT mice displayed no change in latency to fall in a hang wire test, suggesting intact grip strength (Figure 4B). Conditional expression of TDP-43-WT mainly in the forebrain of bigenic mice resulted in mild (22%) incidence of clasping abnormalities, without reaching significance (two-tailed Fisher exact test, *p* = 0.1081; Figure 4C).

**Figure 5:**
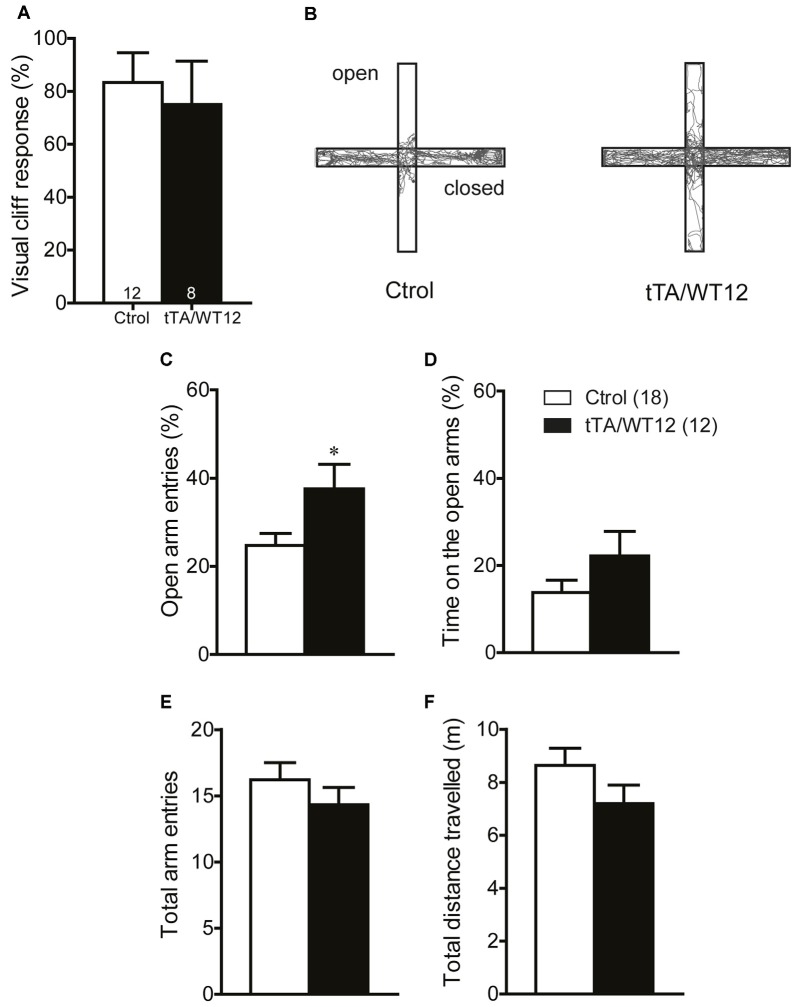
**TDP-43-WT bigenic mice display normal visual perception and signs of decreased anxiety. (A)** Visual perception. Percentage of animals stopping at the edge in the visual cliff test. No significant differences in the response to the edge were found between control and bigenic animals (*p* > 0.05, Mann-Whitney *U* test). **(B–F)** Elevated plus maze test. Mice were placed at the center and allowed to explore the maze freely for 5 min. A mild decrease in anxiety-related behavior was found in bigenic mice. **(B)** Representative track plot. **(C)** Relative open arms entries (*p < 0.05 significantly different from control group, Student’s *t* test). No difference between groups was found in **(D)** percentage of time on open arms, **(E)** total arms entries and **(F)** total distance traveled. (*p* > 0.05 in **D–F**, Student’s *t* test). Number of animals is indicated in parentheses or inside plot bars. Data represent mean ± SEM.

It has been previously reported that cognitive and social performance can be negatively affected due to increased anxiety or sensory deficits (Kazdoba et al., 2016). Importantly, we found no abnormalities in visual function as assessed by the visual cliff test (Figure 5A). In order to assess both anxiety levels and exploratory activity in TDP-43 WT mice, we evaluated performance in the elevated plus maze test. This task presents a conflict between the natural tendency of mice to explore a novel environment and the aversive properties of a brightly lit, open area (Lister, 1987). TDP-43-WT mice displayed a significant increase in the percentage of open arm entries (Student’s *t* test, *t*_(28)_ = 2.280 *p* = 0.0304; Figures 5B,C) and a non-significant trend towards increased percentage of time in the open arms (Student’s *t* test, *t*_(28)_ = 1.459 *p* = 0.1557, Figure 5D), suggesting decreased anxiety-like behavior compared to the control group. Consistent with the lack of altered locomotion in the open field test (Figures 3A,C), the total number of arm entries and the total distance traveled did not differ between groups (Figures 5E,F). These data argues against an unspecific effect on non-motor behaviors due to increased anxiety or impaired visual function. Additionally, they suggest that TDP-43-WT mice may show disinhibition, as reported in other mouse models for FTD (Yin et al., 2010; Ke et al., 2015; Przybyla et al., 2016).

### Time-Dependent Motor Phenotypes Emerge After Prolonged hTDP-43 Overexpression

**Figure 6:**
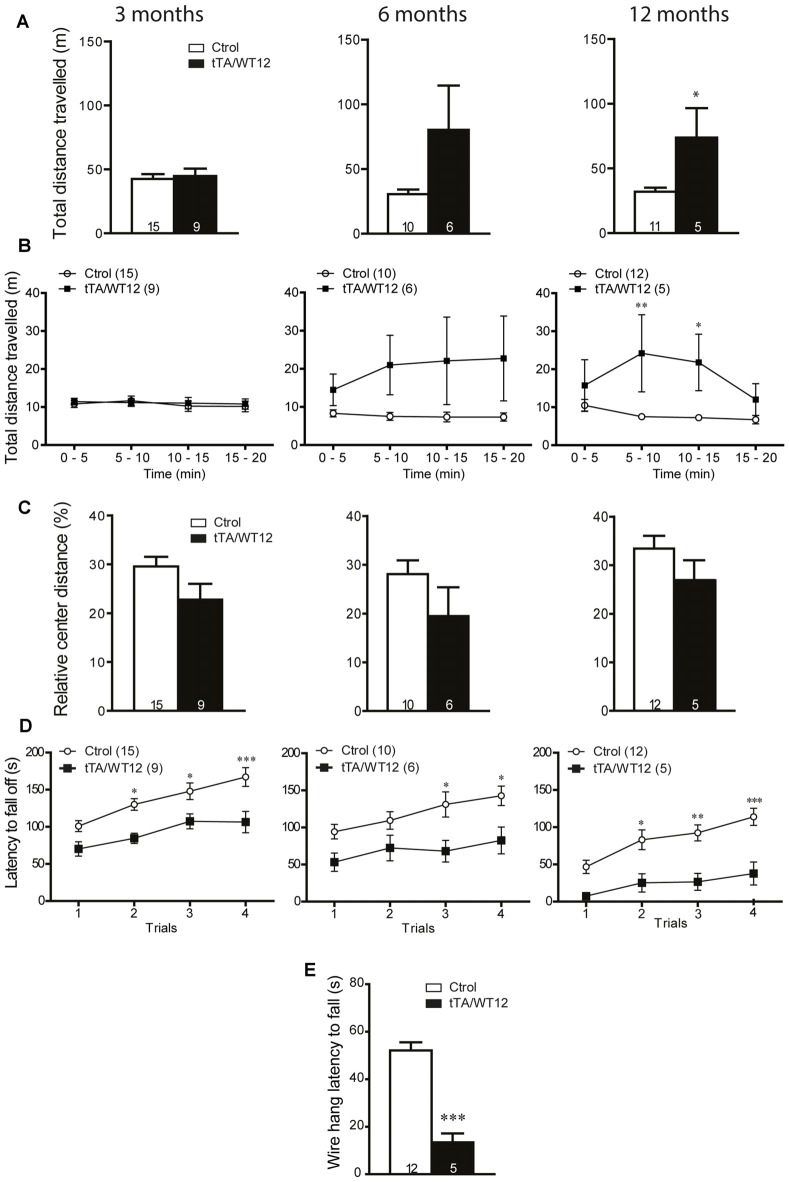
**Time-dependent appearance of motor deficits in TDP-43-WT mice. (A–D)** Motor behavior was analyzed at 3, 6 and 12 months off Dox. **(A–C)** In order to assess general exploratory locomotion in a novel environment, open field test were performed. Mice were placed in a clear (40 cm × 40 cm × 40 cm) arena, and a 20 min session was used.** (A)** Total distance traveled in the open field chamber. An increased trend at 6 months became significant at 12 months of Tg expression (*p < 0.05 significantly different from control group, Student’s *t* test). **(B)** Open field time bin (segments of 5 min) analysis of total distance traveled show a significant difference at 12 months after Tg expression (*p < 0.05; **p < 0.01 significantly different from control group, repeated-measures two-way ANOVA/Bonferroni *post hoc* test). **(C)** Relative center distance during the open field session show no significant differences at any time post Tg induction. **(D)** Accelerated rotarod test. TDP-43-WT mice display impaired coordination and balance at 3, 6 and 12 months after Tg induction (*p < 0.05; **p < 0.01; ***p < 0.001 significantly different from control group, repeated-measures two-way ANOVA/Bonferroni *post hoc* test). **(E)** Grip strength was evaluated at 12 months after Tg expression using the hanging wire grip test. The latency to fall off the wire lid was quantified using a 60 s cutoff time. TDP-43-WT mice show significant deficits in grip strength (***p < 0.001 significantly different from control group, Student’s *t* test). Number of animals is indicated in parentheses or inside plot bars. Data represent mean ± SEM.

The early onset of social and cognitive symptoms with essentially intact motor function displayed by these Tg mice prompted us to analyze if there was a time-dependent decline in motor behavior, reflecting different sensitivity for TDP-43-WT overexpression in brain circuits underlying diverse behavioral domains. We analyzed TDP-43-WT and control littermates at 3, 6 and 12 months post induction (off Dox) in the open field test, and the total distance traveled showed a progressive increase starting at 6 months (Student’s *t* test, *t*_(14)_ = 1.890 *p* = 0.0797), reaching significance 12 months post Tg induction (Figure 6A). Time bin analysis of this parameter also showed a departure from the average levels of control animals after 6 months of Tg expression that did not reach significance, becoming significant at 12 months off Dox (two way repeated-measures ANOVA/Bonferroni’s *post hoc* test, *F*_(1,14)_ = 7.296, *p* = 0.0172 for group; *F*_(3,42)_ = 2.208, *p* = 0.1012 for time; *F*_(3,42)_ = 2.550, *p* = 0.0685 for interaction; Figure 6B). However, the relative center distance remained unchanged at all the post-induction time points evaluated (Figure 6C). In the accelerated rotarod, TDP-43-WT Tg mice demonstrated a poorer performance than controls after 3, 6 and 12 months of Tg induction, displaying a trend toward worse latencies over Tg expression time (two way repeated-measures ANOVA/Bonferroni’s *post hoc* test, for 3 months: *F*_(1,22)_ = 14.83, *p* = 0.0009 for group; *F*_(3,66)_ = 15.11, *p* < 0.0001 for time; *F*_(3,66)_ = 1.134, *p* = 0.3418 for interaction; for 6 months: *F*_(1,14)_ = 7.720, *p* = 0.0148 for group; *F*_(3,42)_ = 7.153, *p* = 0.0005 for time; *F*_(3,42)_ = 1.164, *p* = 0.3349 for interaction; for 12 months: *F*_(1,15)_ = 14.14, *p* = 0.0018 for group; *F*_(3,45)_ = 11.00, *p* < 0.0001 for time; *F*_(3,45)_ = 1.617, *p* = 0.1987 for interaction; Figure 6D). Lastly, the hanging wire test at 12 months off Dox showed profound deficits in grip strength (Student’s *t test*, *t*_(15)_ = 6.551 *p* < 0.0001; Figure 6E), which was normal after 1 month of overexpression (Figure 4B). As a whole, these results demonstrate a progressive motor phenotype in tTA/WT12 mice.

## Discussion

In the present study, we took advantage of a mouse model expressing wild-type hTDP-43 under a system that allows for temporal and regional control of Tg expression. In this way, we were able to analyze multiple domains of the animal behavioral repertoire which could be obscured by the use of constitutive and/or pan-neuronal promoters utilized in other Tg animals modeling the FTD/ALS spectrum of disease.

The main findings from our study are as follows. First, post-weaning overexpression of wild-type hTDP-43 leads to a rapid installment of impairments in social behavior, a characteristic feature of behavioral variant FTD (bvFTD) patients, which is the most common clinical subtype of FTD (Seltman and Matthews, 2012; Laforce, 2013). Second, tTA/WT12 mice display early deficits in cognitive function, most remarkably impaired working memory, a frontal cortex-dependent function. They also show signs of a mild decrease in anxiety in the elevated plus maze test, which some authors interpret as disinhibition and might indicate alterations in amygdala or prefrontal cortex function (Roberson, 2012; Koss et al., 2016). These phenotypes are consistent with those observed in other FTD mouse models (Takeuchi et al., 2011; Przybyla et al., 2016; Vernay et al., 2016a), although in some FTD models an increase in anxiety was also reported (Koss et al., 2016; Liu et al., 2016). Further analysis are required to clearly define if some form of disinhibition is present in tTA/WT12 mice. Third, there is a surprising preservation of motor function at the early time point post-induction (1 month off Dox) when cognitive and social deficits are manifest. Most TDP-43 mouse models present with robust, rapid motoric phenotypes, regardless of appearance of cognitive deficits, and most likely this is at least in part related to the use of pan-neuronal promoters (Tsao et al., 2012; Liu et al., 2013; Philips and Rothstein, 2015). Fourth, we studied in these mice the time course of motor function and identified a progressive appearance of abnormalities including hyperlocomotion, loss of coordination/balance and decreased grip strength.

In the past few years, there has been enormous activity and effort applied to animal model development for FTD/ALS, making good use of the information from recent genetic and neuropathological findings (Roberson, 2012; Liu et al., 2013; Philips and Rothstein, 2015). Specifically, Tg mice based in modulating the expression of C9ORF72, progranulin (PRGN), VCP, CHMP2B and other genes are being generated at a swift pace, and are available for comparison with the variety of TDP-43 based rodent models. In this context, it is becoming more and more difficult to parse out commonalities and differences in the associated neuropathological and behavioral changes. The unique combination of early social/cognitive deficits without motor involvement, which emerges later on with extended periods of TDP-43 expression, suggest that tTA/WT12 mice provide an interesting platform to perform behavioral studies combined with pharmacological approaches difficult to design in other Tg models of ALS/FTD.

In light of the behavioral results reported in this work, we consider that it is relevant to compare them with those observed in a closely related mouse model, termed for short tTA/ΔNLS. This Tg mouse model, developed in parallel with the tTA/WT12 mice (Igaz et al., 2011) and recently behaviorally analyzed (Alfieri et al., 2014), use the same combination of promoter (CamKIIα) and Dox-regulated expression system as the one used in the current study, but with a mutant form of TDP-43 that is cytoplasmically localized due to mutation of the nuclear localization sequence (NLS) in the protein (hTDP-43-ΔNLS). tTA/ΔNLS mice displayed a similar -although more aggressive- degenerative, behavioral and transcriptional phenotype (Igaz et al., 2011; Alfieri et al., 2014). We demonstrate here that tTA/WT12 mice show early (1 month post-weaning Tg induction) social and cognitive deficits, while tTA/ΔNLS mice also rapidly developed a penetrant and florid motor phenotype, which nonetheless allowed the mice to perform most behavioral tasks appropriately, as indicated by control experiments (Alfieri et al., 2014). The reasons for this interesting difference are less than clear at this point, but since both animal models share the same promoter system there should be additional sources for this divergence. One relevant underlying cause might be the different impact of elevated hTDP-43 species on gene expression, as evidenced by genome-wide microarray analysis of cortical samples at early time points post Tg induction (Igaz et al., 2011). Transcriptional changes in tTA/WT12 mice were relatively modest, although they did separate from nTg group after principal component analysis, establishing an “intermediate” gene expression signature. On the other hand, tTA/ΔNLS mice showed >4700 differentially expressed genes respect to nTg mice. This robust alteration in the transcriptional profile of tTA/ΔNLS mice was recently corroborated by RNA-seq analysis, although that study did not analyze tTA/WT12 mice (Amlie-Wolf et al., 2015). Alternative explanations for the different early motor phenotype include the difference in percentage of cells expressing either Tg form (lower in tTA/WT12 mice) and total expression levels of the Tg protein (Igaz et al., 2011), higher in tTA/ΔNLS probably due to override of endogenous autoregulatory mechanisms that govern nuclear TDP-43 levels (Ayala et al., 2011; Budini and Buratti, 2011).

Although many behavioral studies focus predominantly in motor dysfunction, other animal models have shown cognitive and social deficits consistent mostly with the FTD end of the FTD/ALS disease spectrum, i.e., those based in FTD-associated genes including tau, PRGN, CHMP2B and others. The first description of social dysfunction in a TDP-43 based rodent model was our study of behavioral phenotypes in tTA/ΔNLS mice (Alfieri et al., 2014). PRGN deficiency has been used to model PGRN haploinsufficiency-related FTD, and these mice display early social and cognitive phenotypes (Ghoshal et al., 2012; Filiano et al., 2013). A recently described Tg mouse line expressing the human CHMP2B^intron5^ mutant in a neuron specific manner progressively developed FTD-relevant behavioral modifications such as disinhibition, stereotypies, decrease in social interactions and compulsivity (Vernay et al., 2016b). In this context, our behavioral data from tTA/WT12 mice constitutes further evidence for a link between TDP-43 dysregulation and sociability. A forebrain specific, human mutated Tau (hTauP301L + R406W) knock-in mouse showed FTD-relevant phenotypes related to semantic memory, anxiety, anhedonia, sleep and activity (Koss et al., 2016), and mice expressing human P301S mutant tau protein recapitulated neurological deficits of human tauopathies, including early abnormalities in the open field test, the elevated plus-maze test, Y-maze test, Barnes maze test and the Morris water maze test (Takeuchi et al., 2011). Importantly, most of these studies were performed when motor deficits were not preeminent, although in some cases detailed characterization was not reported, in contrast to the current analysis of tTA/WT12 mice. In our TDP-43 WT model, it is not clear yet if certain cognitive and social phenotypes are progressively deteriorated over time, although some cognitive tests performed at 1 month post induction indicate they rapidly reach a performance that cannot worsen. Further studies will be required to better understand the dynamics of these behavioral changes, including also those related to anxiety and disinhibition.

The existence of other animal models with early and profound motor dysfunction (which at this point are the majority of rodent models of FTD/ALS) exemplify the usefulness of having available Tg mice with dissociation of social/cognitive phenotype from motor decline as the one described in this study. Of note, Walker et al. (2015) have recently developed a TDP-43-ΔNLS mouse model (termed rNLS8) with regulatable pan-neuronal expression by means of the NEFH promoter coupled with the same tet-Off system used in this study. Although rNLS8 mice rapidly develop neuropathology and motor deficits consistent with changes observed in ALS, early and robust motor decline leading to death within a few weeks after Tg expression precluded the study of cognitive and social phenotypes more commonly associated with FTD (Walker et al., 2015; Spiller et al., 2016). Moreover, as motor abnormalities gradually emerge in tTA/WT12 mice over time, they provide a unique opportunity to study different presentations or stages shown in the clinicopathological spectrum of human FTD/ALS associated with TDP-43 dysfunction.

Another point that should be noted is that, although relatively scarce, there is evidence that modulating TDP-43 levels changes specific aspects of the biochemical, morphological and electrophysiological properties of neurons. Some authors propose that TDP-43 is an activity-related factor, suggesting roles in mRNA transport and translation in dendrites (Wang et al., 2008) and axons (Alami et al., 2014). There is also recent evidence that hyperexcitability of somatostatin interneurons in TDP-43(A315T) mice contribute to excitotoxicity (Zhang et al., 2016). TDP-43 might alter neuronal morphology and connectivity via regulation of both spinogenesis and neurite outgrowth through small GTPases Rac1 and Rho, respectively (Iguchi et al., 2009; Majumder et al., 2012). Constitutive CamKIIα-TDP-43 mice display attenuated long-term potentiation and decreased levels of plasticity-associated proteins or phosphorylation events, which are concomitant to motor and cognitive phenotypes (Tsai et al., 2010). Besides the functional implications of TDP-43 overexpression, there might be differences in sensitivity to cell death in the neurons composing the brain circuits underlying the different types of behaviors. Further studies investigating these possibilities are warranted.

In summary, we have behaviorally characterized a new conditional mouse model with neuronal expression of hTDP-43 and defined time windows to study early social and cognitive deficits in the absence of overt motor dysfunction, which could better model aspects of “pure” FTD. With progression of Tg expression over time, motor abnormalities become established, giving rise to phenotypes more related to FTD with motor neuron disease. These unique pattern of phenotypic evolution suggest that these mice might be useful to study mechanisms underlying pathological changes associated with TDP-43 proteinopathies.

## Author Contributions

JAA carried out the motor, social, cognitive behavior experiments in young mice, analyzed the data, discussed the results and helped to write the manuscript. PRS performed motor behavior tests in older mice and analyzed data. LMI conceived the project, wrote the manuscript and supervised all aspects of the project.

## Funding

This work was supported by research grants to LMI from Agencia Nacional de Promoción Científica y Tecnológica (ANPCyT) (PICT-PRH 2009-0073 and PICT 2011-1727), the International Brain Research Organization (IBRO Return Home Fellowship 2009), Fundación Florencio Fiorini, Fundación Alberto Roemmers and the University of Buenos Aires (UBACyT; grant no. 20020110200160). LMI is a member of Consejo Nacional de Investigaciones Científicas y Técnicas (CONICET). JAA and PRS were supported by doctoral fellowships from CONICET.

## Conflict of Interest Statement

The authors declare that the research was conducted in the absence of any commercial or financial relationships that could be construed as a potential conflict of interest.

## References

[B1] Alami N. H. Smith R. B. Carrasco M. A. Williams L. A. Winborn C. S. Han S. S.. (2014). Axonal transport of TDP-43 mRNA granules is impaired by ALS-causing mutations. Neuron 81, 536–543. 10.1016/j.neuron.2013.12.018 24507191 PMC3939050

[B2] Alfieri J. A. Pino N. S. Igaz L. M. (2014). Reversible behavioral phenotypes in a conditional mouse model of TDP-43 proteinopathies. J. Neurosci. 34, 15244–15259. 10.1523/JNEUROSCI.1918-14.2014 25392493 PMC4298649

[B3] Amlie-Wolf A. Ryvkin P. Tong R. Dragomir I. Suh E. Xu Y.. (2015). Transcriptomic changes due to cytoplasmic TDP-43 expression reveal dysregulation of histone transcripts and nuclear chromatin. PLoS One 10:e0141836. 10.1371/journal.pone.0141836 26510133 PMC4624943

[B4] Ayala Y. M. De Conti L. Avendaño-Vázquez S. E. Dhir A. Romano M. D’Ambrogio A.. (2011). TDP-43 regulates its mRNA levels through a negative feedback loop. EMBO J. 30, 277–288. 10.1038/emboj.2010.310 21131904 PMC3025456

[B5] Baralle M. Buratti E. Baralle F. E. (2013). The role of TDP-43 in the pathogenesis of ALS and FTLD. Biochem. Soc. Trans. 41, 1536–1540. 10.1042/BST20130186 24256250

[B6] Belforte J. E. Zsiros V. Sklar E. R. Jiang Z. Yu G. Li Y.. (2010). Postnatal NMDA receptor ablation in corticolimbic interneurons confers schizophrenia-like phenotypes. Nat. Neurosci. 13, 76–83. 10.1038/nn.2447 19915563 PMC2797836

[B7] Boccia M. M. Acosta G. B. Blake M. G. Baratti C. M. (2004). Memory consolidation and reconsolidation of an inhibitory avoidance response in mice: effects of i.c.v. injections of hemicholinium-3. Neuroscience 124, 735–741. 10.1016/j.neuroscience.2004.01.001 15026114

[B8] Braz B. Y. Galiñanes G. L. Taravini I. R. Belforte J. E. Murer M. G. (2015). Altered corticostriatal connectivity and exploration/exploitation imbalance emerge as intermediate phenotypes for a neonatal dopamine dysfunction. Neuropsychopharmacology 40, 2576–2587. 10.1038/npp.2015.104 25872916 PMC4569947

[B9] Brodkin E. S. Hagemann A. Nemetski S. M. Silver L. M. (2004). Social approach-avoidance behavior of inbred mouse strains towards DBA/2 mice. Brain Res. 1002, 151–157. 10.1016/j.brainres.2003.12.013 14988045

[B10] Budini M. Buratti E. (2011). TDP-43 autoregulation: implications for disease. J. Mol. Neurosci. 45, 473–479. 10.1007/s12031-011-9573-8 21681666

[B11] Ferrari R. Kapogiannis D. Huey E. D. Momeni P. (2011). FTD and ALS: a tale of two diseases. Curr. Alzheimer Res. 8, 273–294. 10.2174/156720511795563700 21222600 PMC3801195

[B12] Filiano A. J. Martens L. H. Young A. H. Warmus B. A. Zhou P. Diaz-Ramirez G.. (2013). Dissociation of frontotemporal dementia-related deficits and neuroinflammation in progranulin haploinsufficient mice. J. Neurosci. 33, 5352–5361. 10.1523/JNEUROSCI.6103-11.2013 23516300 PMC3740510

[B13] Gascon E. Lynch K. Ruan H. Almeida S. Verheyden J. M. Seeley W. W.. (2014). Alterations in microRNA-124 and AMPA receptors contribute to social behavioral deficits in frontotemporal dementia. Nat. Med. 20, 1444–1451. 10.1038/nm.3717 25401692 PMC4257887

[B14] Ghoshal N. Dearborn J. T. Wozniak D. F. Cairns N. J. (2012). Core features of frontotemporal dementia recapitulated in progranulin knockout mice. Neurobiol. Dis. 45, 395–408. 10.1016/j.nbd.2011.08.029 21933710 PMC3225509

[B15] Giordana M. T. Ferrero P. Grifoni S. Pellerino A. Naldi A. Montuschi A. (2011). Dementia and cognitive impairment in amyotrophic lateral sclerosis: a review. Neurol. Sci. 32, 9–16. 10.1007/s10072-010-0439-6 20953810

[B16] Gordon P. H. (2013). Amyotrophic lateral sclerosis: an update for 2013 clinical features, pathophysiology, management and therapeutic trials. Aging Dis. 4, 295–310. 10.14336/AD.2013.0400295 24124634 PMC3794725

[B17] Harciarek M. Cosentino S. (2013). Language, executive function and social cognition in the diagnosis of frontotemporal dementia syndromes. Int. Rev. Psychiatry 25, 178–196. 10.3109/09540261.2013.763340 23611348 PMC4481322

[B18] Hodges J. R. Davies R. R. Xuereb J. H. Casey B. Broe M. Bak T. H.. (2004). Clinicopathological correlates in frontotemporal dementia. Ann. Neurol. 56, 399–406. 10.1002/ana.20203 15349867

[B19] Igaz L. M. Kwong L. K. Lee E. B. Chen-Plotkin A. Swanson E. Unger T.. (2011). Dysregulation of the ALS-associated gene TDP-43 leads to neuronal death and degeneration in mice. J. Clin. Invest. 121, 726–738. 10.1172/JCI44867 21206091 PMC3026736

[B20] Iguchi Y. Katsuno M. Niwa J.-I. Yamada S.-I. Sone J. Waza M.. (2009). TDP-43 depletion induces neuronal cell damage through dysregulation of Rho family GTPases. J. Biol. Chem. 284, 22059–22066. 10.1074/jbc.M109.012195 19535326 PMC2755930

[B21] Kazdoba T. M. Leach P. T. Crawley J. N. (2016). Behavioral phenotypes of genetic mouse models of autism. Genes Brain Behav. 15, 7–26. 10.1111/gbb.12256 26403076 PMC4775274

[B22] Ke Y. D. van Hummel A. Stevens C. H. Gladbach A. Ippati S. Bi M.. (2015). Short-term suppression of A315T mutant human TDP-43 expression improves functional deficits in a novel inducible transgenic mouse model of FTLD-TDP and ALS. Acta Neuropathol. 130, 661–678. 10.1007/s00401-015-1486-0 26437864

[B23] Koss D. J. Robinson L. Drever B. D. Plucinska K. Stoppelkamp S. Veselcic P.. (2016). Mutant Tau knock-in mice display frontotemporal dementia relevant behaviour and histopathology. Neurobiol. Dis. 91, 105–123. 10.1016/j.nbd.2016.03.002 26949217

[B24] Kwong L. K. Uryu K. Trojanowski J. Q. Lee V. M. (2008). TDP-43 proteinopathies: neurodegenerative protein misfolding diseases without amyloidosis. Neurosignals 16, 41–51. 10.1159/000109758 18097159

[B25] Laforce R. Jr. (2013). Behavioral and language variants of frontotemporal dementia: a review of key symptoms. Clin. Neurol. Neurosurg. 115, 2405–2410. 10.1016/j.clineuro.2013.09.031 24446563

[B26] Lalonde R. (2002). The neurobiological basis of spontaneous alternation. Neurosci. Biobehav. Rev. 26, 91–104. 10.1016/s0149-7634(01)00041-0 11835987

[B27] Lister R. G. (1987). The use of a plus-maze to measure anxiety in the mouse. Psychopharmacology 92, 180–185. 10.1007/bf00177912 3110839

[B29] Liu Y. C. Chiang P. M. Tsai K. J. (2013). Disease animal models of TDP-43 proteinopathy and their pre-clinical applications. Int. J. Mol. Sci. 14, 20079–20111. 10.3390/ijms141020079 24113586 PMC3821604

[B28] Liu Y. Pattamatta A. Zu T. Reid T. Bardhi O. Borchelt D. R.. (2016). C9orf72 BAC mouse model with motor deficits and neurodegenerative features of ALS/FTD. Neuron 90, 521–534. 10.1016/j.neuron.2016.04.005 27112499

[B30] Majumder P. Chen Y. T. Bose J. K. Wu C. C. Cheng W. C. Cheng S. J.. (2012). TDP-43 regulates the mammalian spinogenesis through translational repression of Rac1. Acta Neuropathol. 124, 231–245. 10.1007/s00401-012-1006-4 22760527

[B31] Mayford M. Bach M. E. Huang Y. Y. Wang L. Hawkins R. D. Kandel E. R. (1996). Control of memory formation through regulated expression of a CaMKII transgene. Science 274, 1678–1683. 10.1126/science.274.5293.1678 8939850

[B32] Morris J. (2015). Amyotrophic Lateral Sclerosis (ALS) and related motor neuron diseases: an overview. Neurodiagn. J. 55, 180–194. 10.1080/21646821.2015.1075181 26630810

[B33] Neumann M. Sampathu D. M. Kwong L. K. Truax A. C. Micsenyi M. C. Chou T. T.. (2006). Ubiquitinated TDP-43 in frontotemporal lobar degeneration and amyotrophic lateral sclerosis. Science 314, 130–133. 10.1126/science.1134108 17023659

[B34] Philips T. Rothstein J. D. (2015). Rodent models of amyotrophic lateral sclerosis. Curr. Protoc. Pharmacol. 69, 5.67.1–5.67.21. 10.1002/0471141755.ph0567s69 26344214 PMC4562058

[B35] Picher-Martel V. Valdmanis P. N. Gould P. V. Julien J. P. Dupre N. (2016). From animal models to human disease: a genetic approach for personalized medicine in ALS. Acta Neuropathol. Commun. 4:70. 10.1186/s40478-016-0340-5 27400686 PMC4940869

[B36] Przybyla M. Stevens C. H. van der Hoven J. Harasta A. Bi M. Ittner A.. (2016). Disinhibition-like behavior in a P301S mutant tau transgenic mouse model of frontotemporal dementia. Neurosci. Lett. 631, 24–29. 10.1016/j.neulet.2016.08.007 27521751

[B37] Roberson E. D. (2012). Mouse models of frontotemporal dementia. Ann. Neurol. 72, 837–849. 10.1002/ana.23722 23280835 PMC3539234

[B38] Seltman R. E. Matthews B. R. (2012). Frontotemporal lobar degeneration: epidemiology, pathology, diagnosis and management. CNS Drugs 26, 841–870. 10.2165/11640070-000000000-00000 22950490

[B39] Shinagawa S. Ikeda M. Fukuhara R. Tanabe H. (2006). Initial symptoms in frontotemporal dementia and semantic dementia compared with Alzheimer’s disease. Dement. Geriatr. Cogn. Disord. 21, 74–80. 10.1159/000090139 16340203

[B40] Spiller K. J. Restrepo C. R. Khan T. Stieber A. M. Kwong L. K. Trojanowski J. Q.. (2016). Progression of motor neuron disease is accelerated and the ability to recover is compromised with advanced age in rNLS8 mice. Acta Neuropathol. Commun. 4:105. 10.1186/s40478-016-0377-5 27687289 PMC5043606

[B41] Takeuchi H. Iba M. Inoue H. Higuchi M. Takao K. Tsukita K.. (2011). P301S mutant human tau transgenic mice manifest early symptoms of human tauopathies with dementia and altered sensorimotor gating. PLoS One 6:e21050. 10.1371/journal.pone.0021050 21698260 PMC3115982

[B42] Tsai K. J. Yang C. H. Fang Y. H. Cho K. H. Chien W. L. Wang W. T.. (2010). Elevated expression of TDP-43 in the forebrain of mice is sufficient to cause neurological and pathological phenotypes mimicking FTLD-U. J. Exp. Med. 207, 1661–1673. 10.1084/jem.20092164 20660618 PMC2916125

[B43] Tsao W. Jeong Y. H. Lin S. Ling J. Price D. L. Chiang P. M.. (2012). Rodent models of TDP-43: recent advances. Brain Res. 1462, 26–39. 10.1016/j.brainres.2012.04.031 22608070 PMC3613131

[B44] Vernay A. Sellal F. René F. (2016a). Evaluating behavior in mouse models of the behavioral variant of frontotemporal dementia: which test for which symptom? Neurodegener. Dis. 16, 127–139. 10.1159/000439253 26517704

[B45] Vernay A. Therreau L. Blot B. Risson V. Dirrig-Grosch S. Waegaert R.. (2016b). A transgenic mouse expressing CHMP2B^intron5^ mutant in neurons develops histological and behavioural features of amyotrophic lateral sclerosis and frontotemporal dementia. Hum. Mol. Genet. [Epub ahead of print]. 10.1093/hmg/ddw182 27329763

[B46] Walker A. K. Spiller K. J. Ge G. Zheng A. Xu Y. Zhou M.. (2015). Functional recovery in new mouse models of ALS/FTLD after clearance of pathological cytoplasmic TDP-43. Acta Neuropathol. 130, 643–660. 10.1007/s00401-015-1460-x 26197969 PMC5127391

[B47] Wang I. F. Wu L. S. Chang H. Y. Shen C. K. (2008). TDP-43, the signature protein of FTLD-U, is a neuronal activity-responsive factor. J. Neurochem. 105, 797–806. 10.1111/j.1471-4159.2007.05190.x 18088371

[B48] Warburton E. C. Brown M. W. (2010). Findings from animals concerning when interactions between perirhinal cortex, hippocampus and medial prefrontal cortex are necessary for recognition memory. Neuropsychologia 48, 2262–2272. 10.1016/j.neuropsychologia.2009.12.022 20026141

[B49] Woollacott I. O. Rohrer J. D. (2016). The clinical spectrum of sporadic and familial forms of frontotemporal dementia. J. Neurochem. 138, 6–31. 10.1111/jnc.13654 27144467

[B50] Yin F. Dumont M. Banerjee R. Ma Y. Li H. Lin M. T.. (2010). Behavioral deficits and progressive neuropathology in progranulin-deficient mice: a mouse model of frontotemporal dementia. FASEB J. 24, 4639–4647. 10.1096/fj.10-161471 20667979 PMC2992364

[B51] Zhang W. Zhang L. Liang B. Schroeder D. Zhang Z. W. Cox G. A.. (2016). Hyperactive somatostatin interneurons contribute to excitotoxicity in neurodegenerative disorders. Nat. Neurosci. 19, 557–559. 10.1038/nn.4257 26900927 PMC4811704

